# Speckle Tracking Analysis of the Left and Right Heart in Type 1 Diabetes Patients with End-Stage Renal Disease Awaiting Simultaneous Pancreas–Kidney Transplantation

**DOI:** 10.3390/diagnostics16193166

**Published:** 2026-09-29

**Authors:** Kun Zhang, Katharina Haag, Ingo Hilgendorf, Fabian Halleck, Andreas Kahl, Lukas J. Lehner

**Affiliations:** 1 Department of Cardiology, Angiology and Intensive Care Medicine, Deutsches Herzzentrum der Charité, Campus Virchow-Klinikum, 13353 Berlin, Germany; 2 DZHK (German Centre for Cardiovascular Research), Partner Site Berlin, 10785 Berlin, Germany; 3 Department of Nephrology and Medical Intensive Care, Charité—Universitätsmedizin Berlin, 13353 Berlin, Germany; 4 Department of Radiology, Charité—Universitätsmedizin Berlin, 10117 Berlin, Germany

**Keywords:** diabetic cardiomyopathy, speckle tracking echocardiography, strain, diabetes mellitus type 1, pancreas–kidney transplantation, end-stage renal disease

## Abstract

**Background/Objectives:** Diabetic cardiomyopathy is an under-recognized complication of type 1 diabetes mellitus (T1DM), often co-existing with diabetic nephropathy. This study investigates whether patients with T1DM and end-stage renal disease (ESRD) undergoing simultaneous pancreas–kidney transplantation (SPKT) show echocardiographic signs of cardiomyopathy prior to surgery and explores whether cardiac changes are more attributable to diabetes itself or to chronic kidney disease. **Methods**: We retrospectively analyzed preoperative echocardiographic data of 16 SPKT patients with T1DM and ESRD, using 2D speckle tracking for strain assessment. Data were compared to individually matched control groups: (1) ESRD patients without diabetes (*n* = 20) and (2) healthy controls (*n* = 48). Left and right ventricular/atrial function and structure were analyzed, including 2D speckle-tracking analysis. A mixed-effects model adjusted for age, sex, coronary artery disease, and hypertension. **Results:** SPKT patients exhibited significant structural changes in the left heart, including increased left ventricular mass and left atrial volume index, alongside reduced global longitudinal strain (GLS), global radial strain (GRS), and global circumferential strain (GCS) compared to healthy controls. GRS and GCS were significantly lower in the SPKT group than in the ESRD group without diabetes. Left atrial strain and strain rates were reduced in both disease groups. Right ventricular and atrial strain parameters did not significantly differ between groups. Mixed model analysis confirmed significant strain differences in the left ventricle even after adjusting for confounders. **Conclusions:** T1DM patients with end-stage nephropathy awaiting SPKT show echocardiographic abnormalities compatible with subclinical myocardial dysfunction, with predominant involvement of the left heart. Compared to non-diabetic ESRD patients, the diabetic cohort exhibited greater impairment in left ventricular strain parameters, supporting a pathophysiologic role of diabetes independent of kidney disease. Speckle-tracking analysis demonstrated potential utility for detecting early signs of myocardial dysfunction in this high-risk population.

## 1. Introduction

Diabetes mellitus type 1 (T1DM) is a chronic autoimmune disease marked by insulin deficiency and associated with high morbidity due to long-term microvascular complications [1]. Diabetic nephropathy affects approximately 30–40% of patients with T1DM and remains a leading cause of end-stage renal disease (ESRD) [2]. In selected severe cases, simultaneous pancreas–kidney transplantation (SPKT) is considered the optimal therapeutic approach, aiming to restore both, normoglycemia and kidney function [3,4].

A less understood, but increasingly recognized complication of T1DM is diabetic cardiomyopathy, a primary myocardial disease independent of hypertension, coronary artery disease, or valvular abnormalities. It is characterized by concentric left ventricular hypertrophy, interstitial fibrosis, and diastolic or systolic dysfunction, with preserved ejection fraction in early stages [5,6,7,8,9].

Cardiovascular disease remains the leading cause of death in diabetic patients, and emerging evidence suggests that myocardial dysfunction in diabetic cardiomyopathy may begin long before symptoms or traditional echocardiographic abnormalities become apparent. Two-dimensional speckle tracking echocardiography has emerged as a powerful technique to detect subclinical myocardial impairment by analyzing myocardial deformation in multiple directions, including global longitudinal strain (GLS), circumferential strain (GCS), and radial strain (GRS) [10,11].

However, the specific contribution of diabetes versus uremia in the development of myocardial dysfunction remains unclear, particularly in the context of T1DM patients with diabetic nephropathy awaiting SPKT [12,13,14]. This study aims to evaluate echocardiographic and speckle tracking findings in SPKT candidates and compare them with matched controls with ESRD but no diabetes, and healthy individuals, to differentiate diabetic from uremic cardiomyopathy.

## 2. Methods

This retrospective observational study was conducted at the Department of Cardiology, Charité Campus Virchow-Klinikum, Berlin. The study protocol conforms to the ethical guidelines of the 1975 Declaration of Helsinki. This study was approved by the local ethics committee (#EA4/202/19).

We screened all patients who underwent SPKT between 1 January 2005, and 31 December 2018. Only patients with T1DM, ESRD, and a transthoracic echocardiogram with 2D speckle tracking imaging prior to transplantation were included. Out of 335 initial cases, only 16 met all inclusion criteria and formed the SPKT group. The main reason for exclusion was the lack of high-quality echocardiographic raw data, as was frequently the case when cardiac assessment had been performed in the outpatient setting.

Controls were individually matched to SPKT patients according to sex and age (±2 years). Three healthy controls were matched to each SPKT patient, whereas one to two ESRD controls were matched per SPKT patient, resulting in 48 healthy controls and 20 ESRD controls. Given the small size of the SPKT cohort, a higher matching ratio was chosen for the healthy control and ESRD groups to increase statistical precision.

Data were extracted from the SAP and TBase systems, and echocardiograms were analyzed using EchoPAC version 201 (GE Ultrasound, Horten, Norway). Image acquisition was performed by different experienced sonographers as part of routine clinical care with the standard acquisition methods. Strain analyses were performed retrospectively and blinded by the same observer to ensure consistency across the study groups. For quality control, a subset of measurements was independently reviewed by a second experienced observer. No formal assessment of interobserver or intraobserver reproducibility was performed.

Conventional echocardiographic parameters included evaluation of left and right ventricular systolic function, chamber dimensions, and diastolic function. For assessment of left ventricular global longitudinal strain (LV GLS), apical two-, three-, and four-chamber views were used. GCS and GRS were assessed in the parasternal short-axis view at the level of the papillary muscles. Right ventricular global longitudinal strain (RV GLS) was evaluated from the four-chamber view. For the left atrium, peak atrial longitudinal strain (PALS), peak atrial contraction strain (PACS), and atrial strain rates reflecting reservoir (SRs), conduit (SRe), and contractile function (SRa) were assessed from the apical two- and four-chamber views. For the right atrium, the corresponding strain parameters were assessed from the apical four-chamber view. The strain values are given in absolute numbers.

Statistical analysis was performed using SPSS (v. 27). Given the small sample size and variability of several parameters, non-parametric methods were used for continuous group comparisons. Continuous demographic, clinical, follow-up, and echocardiographic variables were compared among the three groups using the Kruskal–Wallis test. In cases of a significant overall group difference, pairwise post hoc comparisons were performed with Bonferroni adjustment for multiple comparisons. Categorical variables were compared using the chi-square test or Fisher’s exact test, as appropriate. Linear mixed-effects models were used to assess differences in strain parameters between the groups. Clinical group, age, sex, hypertension, and coronary artery disease were included as fixed effects, while matching ID was included as a random effect to account for the matched study design. The SPKT group served as the reference category; therefore, the reported β coefficient for clinical group represents the adjusted mean difference between the control and SPKT groups. A *p* value < 0.05 was considered statistically significant.

## 3. Results

A total of 16 patients with T1DM and ESRD who underwent SPKT met all inclusion criteria (SPKT group). The control groups included 20 matched ESRD patients without diabetes and 48 healthy individuals (healthy controls). The mean age across all groups was similar due to matching (mean age 42 years), and there was a balanced sex distribution.

### 3.1. Demographic and Clinical Characteristics

Baseline demographics showed no significant differences between SPKT and ESRD groups in terms of age, sex, hypertension, or coronary artery disease (Table 1). Body mass index (BMI) was slightly lower in the SPKT. All SPKT patients had longstanding T1DM (>20 years) and advanced kidney disease.

### 3.2. Echocardiographic Standard Parameters

Left ventricular (LV) morphology and function (Table 2) showed significant group differences. Patients in the ESRD group had the largest LV dimensions (LVEDD and LVESD), and both SPKT and ESRD patients exhibited significantly increased LV wall thickness (IVS, PW) and LV mass (LVM, LVMI) compared to controls. Diastolic function parameters (E, A, E/A ratio, DT, e′ sept/lat) were significantly impaired in SPKT and ESRD patients, with notably elevated E/e′ ratios, suggesting increased filling pressures. Left ventricular ejection fraction (LVEF) was significantly reduced in SPKT and ESRD patients compared to controls, but still within the range of preserved ejection fraction.

Left atrial (LA) remodeling (Table 3) was more pronounced in both patient groups. LA volumes and indices (LAVol max/pre-a/min and LAVI max/pre-a/min) were significantly higher in SPKT and ESRD patients compared to controls. Notably, LA pre-a length and volume parameters were particularly enlarged in the SPKT group.

Right ventricular (RV) structure and function (Table 4) also differed significantly across groups. RV basal and mid diameters (RVEDD) were significantly larger in both SPKT and ESRD groups compared to controls. RV areas were significantly enlarged in the patient groups. Systolic function parameters such as TAPSE and tissue Doppler velocity (s’) were reduced in SPKT and ESRD groups compared to controls, though fractional area change (FAC) remained preserved across all groups. Estimated pulmonary pressures (TR velocity, sPAP) showed a trend toward elevation in patient groups, but differences did not reach statistical significance.

Right atrial (RA) geometry (Table 5) showed significantly increased RA area, volume, and indexed volume in SPKT and ESRD patients compared to controls, with no differences observed between SPKT and ESRD.

### 3.3. Strain Analysis

Left ventricular strain analysis revealed significantly impaired deformation in both patient groups compared to healthy controls (Table 6). GLS, assessed at basal, mid, and apical segments, was markedly reduced in both patient groups relative to controls (*p* < 0.001 for all), with no significant difference between SPKT and ESRD patients. Average GLS showed the most pronounced impairment in SPKT patients (10.69 ± 2.42%) compared to controls (17.19 ± 1.68%; *p* < 0.001). GCS and GRS were also significantly reduced in both patient groups versus controls (*p* < 0.001). Notably, GCS was lower in SPKT compared to ESRD patients (*p* = 0.002), while GRS was significantly higher in controls than in ESRD patients (*p* = 0.047).

Left atrial strain parameters were significantly impaired in both patient groups (Table 7). PACS was most reduced in the ESRD group (11.57 ± 4.22%) compared to both controls and SPKT patients (*p* = 0.002). PALS, as well as atrial strain rates during systole (SRs), early diastole (SRe), and atrial contraction (SRa), were significantly lower in both patient groups compared to controls (all *p* < 0.05).

Right ventricular strain analysis did not reveal significant differences between groups (Table 8). RV GLS including free wall, endocardial, midmyocardial, and epicardial components were not significantly different across groups.

Right atrial strain analysis (Table 9) revealed impaired atrial contraction strain (SRa) in both patient groups compared to controls. RA strain parameters (PACS, PALS, SRs, SRe) showed no statistically significant differences across groups, although a trend toward reduced reservoir and conduit function was noted in the SPKT and ESRD groups.

### 3.4. Predictors of Left Ventricular Myocardial Strain

To identify independent predictors of impaired myocardial deformation, mixed-effects linear models were constructed for GLS, GCS, and GRS, with clinical group (SPKT/ESRD/control), age, sex, hypertension, and coronary artery disease as covariates.

GLS was significantly associated with clinical group status (β = −5.68, CI: −7.48 to −3.88, *p* < 0.001), indicating that patients with end-stage renal disease—particularly those with diabetes—had substantially reduced longitudinal function. None of the other covariates, including age, sex, hypertension, or coronary artery disease, showed a significant influence on GLS (Figure 1).

GCS was also independently associated with group status (β = −8.51, CI −14.22 to −2.80, *p* = 0.004), confirming impaired circumferential fiber shortening in the patient groups. In addition, female sex was associated with lower GCS values (β = −4.69, 95% CI −8.55 to −0.84, *p* = 0.018), suggesting potential sex-based differences in circumferential strain mechanics. Age, hypertension, and coronary artery disease were not significant predictors in the model (Figure 2).

In contrast, GRS showed a positive association with patient group (β = 14.78, CI 3.90 to 25.66, *p* = 0.009), reflecting higher radial strain values in the ESRD groups. Age was inversely associated with GRS (β = −0.66, CI −1.13 to −0.19, *p* = 0.007), indicating declining radial deformation with increasing age. Other covariates, including sex, hypertension, and coronary artery disease, were not significantly associated with GRS (Figure 3).

## 4. Discussion

This study provides novel insights into myocardial structure and function in a unique cohort of T1DM patients with advanced diabetic nephropathy awaiting SPKT. It presents a detailed echocardiographic analysis comparing patients with T1DM and ESRD awaiting SPKT, patients with ESRD without diabetes, and healthy controls. Using conventional and 2D speckle-tracking echocardiography, we identified subclinical systolic dysfunction in the SPKT cohort, particularly in left ventricular deformation parameters such as GLS, GRS, and GCS, despite preserved LVEF.

Our findings support the hypothesis that diabetes combined with renal failure is associated with greater impairment of systolic myocardial function than renal failure alone, suggesting an additional contribution of diabetes. Although both SPKT and ESRD patients had signs of hypertrophy and atrial enlargement, strain values in the SPKT group were more severely impaired, especially GRS and GCS.

Left heart structural and functional differences were pronounced in both patient groups compared to healthy controls. Although LVEF remained within normal limits, patients in both groups (SPKT and ESRD) showed significantly reduced GLS, GCS, and GRS, suggesting early systolic dysfunction undetectable by conventional metrics. Notably, GCS and GRS were even more impaired in the SPKT group, implying a more advanced stage of myocardial remodeling in these patients, potentially attributable to the combined effects of diabetic and uremic cardiomyopathy.

Concentric left ventricular hypertrophy was observed in both patient groups, with elevated left ventricular mass (LVM), mass index (LVMI), and left ventricular end-diastolic volume (LVEDV) more pronounced in ESRD patients. Left atrial volume index (LAVI) and strain measures (PALS, PACS, strain rates) were also significantly increased or reduced, respectively, particularly in the ESRD group, indicating elevated left ventricular filling pressures due to diastolic dysfunction and ESRD associated hypervolemia. However, despite equally high E/e′ parameters SPKT patients showed smaller LVEDV values, suggesting advanced diastolic dysfunction without evident cardiac hypervolemia These findings indicate a possible additional association of diabetes with cardiac dysfunction beyond the effects of ESRD and volume status. In addition, these results mark the utility of atrial strain as a sensitive marker for early diastolic impairment in patients with diabetes and/or renal disease [15,16].

The early impairment of systolic deformation—especially GLS —prior to measurable changes in LVEF underscores the utility of speckle tracking as a sensitive diagnostic method. These findings are consistent with prior studies demonstrating that GLS deteriorates earlier than conventional EF metrics in diabetes [17,18,19].

Prior studies have demonstrated that both entities, diabetic and uremic cardiomyopathy, are associated with LV hypertrophy, diastolic and systolic dysfunction, and myocardial fibrosis. Subclinical systolic dysfunction is often first detectable through impaired longitudinal strain, particularly in the absence of LVEF changes. Several studies in children and adults with type 1 diabetes have shown significantly impaired GLS and GCS values, even early after diagnosis [20,21]. Similarly, patients with ESRD exhibit early myocardial impairment detectable by strain imaging, regardless of preserved EF [22].

This present study not only confirms that cardiac function is impaired in patients with T1DM and ESRD, but contributes novel evidence that both diabetes and renal disease exert independent and synergistic effects on cardiac mechanics—even in relatively young and asymptomatic patients.

Several pathophysiological mechanisms may potentially contribute to the observed differences and should be considered. The differences between SPKT and ESRD patients may reflect distinct patterns of myocardial remodeling, although these mechanisms cannot be established from the present data. ESRD patients exhibited more pronounced indices of elevated left ventricular filling pressures, which could be related to longer dialysis exposure, chronic uremic remodeling, and differences in volume status. In contrast, SPKT patients showed greater impairment in some systolic strain parameters despite a similar degree of diastolic dysfunction. Longstanding diabetes and associated microvascular complications may represent one possible contributor to these findings. Chronic hyperglycemia has been implicated in several mechanisms that may affect myocardial function, including the accumulation of advanced glycation end-products, oxidative stress, and inflammation, all of which have been associated with myocardial fibrosis, hypertrophy, and apoptosis [23,24,25].

Using a mixed-effects model, we showed that SPKT patients had significantly reduced LV GLS, GCS, and GRS values compared to matched healthy controls, even after adjusting for age, sex, hypertension, and coronary artery disease. Importantly, older age and male sex were independently associated with reduced strain values—consistent with the prior literature. Interestingly, coronary artery disease and hypertension were not significantly associated with strain impairment in this cohort. However, the small number of patients with coronary artery disease and the limited overall sample size reduce the statistical power to reliably assess its independent effect on myocardial strain.

Right heart strain parameters were preserved in both pathological groups, pointing toward a left-dominant pathology. While left atrial strain measures were also reduced, these did not remain significant after multivariable adjustment, indicating confounding effects such as age, sex, and cardiovascular comorbidities.

Limitations of the study include its retrospective, single-center design and small sample size. The rarity of SPKT and the difficulty in obtaining high-quality echocardiographic images limited the number of included patients. Although higher matching ratios were used for the control groups to improve statistical precision, the small size of the SPKT cohort remains an important limitation, particularly for multivariable analyses including infrequent covariates. The retrospective selection of patients based on the availability of adequate pre-transplant echocardiographic data may have introduced selection bias, as included patients may have differed systematically from excluded patients with regard to cardiovascular risk or clinical status. Moreover, echocardiograms were acquired by different operators, introducing potential variability. The lack of detailed information on dialysis timing relative to echocardiography, ultrafiltration volume, and volume status represents a potential source of residual confounding, particularly when interpreting echocardiographic parameters that may be influenced by volume loading.

To our knowledge, this is the first study to systematically evaluate speckle tracking parameters in SPKT candidates. The findings advocate for the inclusion of advanced echocardiographic strain analysis in pre-transplant evaluations, potentially improving risk stratification and therapeutic planning.

## 5. Conclusions

Patients with type 1 diabetes and diabetic nephropathy awaiting simultaneous pancreas–kidney transplantation exhibit early echocardiographic markers of myocardial dysfunction, with findings suggesting an additional association with diabetes beyond the effects of renal dysfunction alone. In particular, deformation parameters such as global longitudinal strain, global radial strain, and global circumferential strain offer enhanced diagnostic sensitivity and should be considered valuable tools for the early detection of subclinical myocardial dysfunction in this high-risk population.

## Figures and Tables

**Figure 1:**
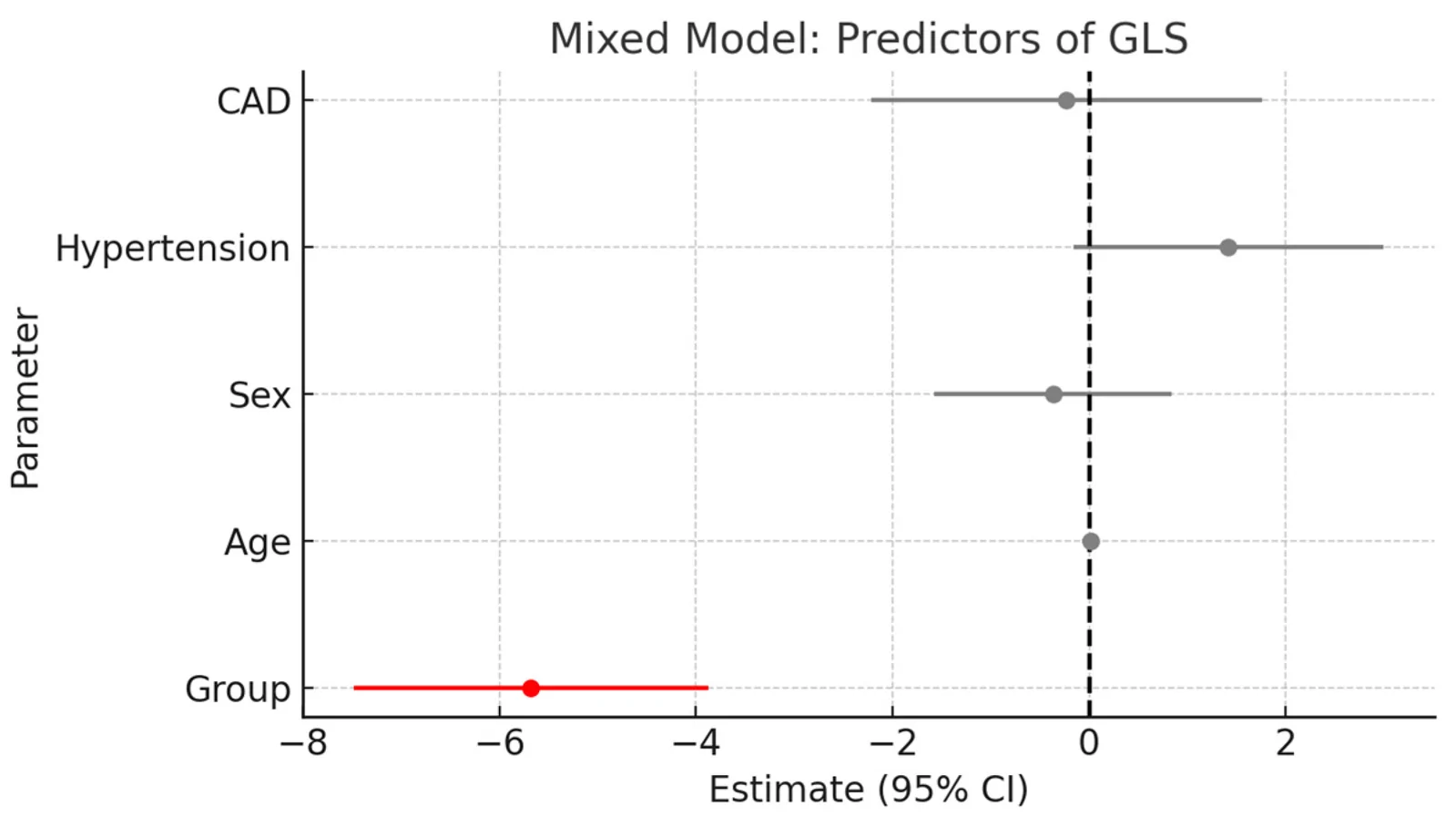
Mixed model: Dependent variable—GLS.

**Figure 2:**
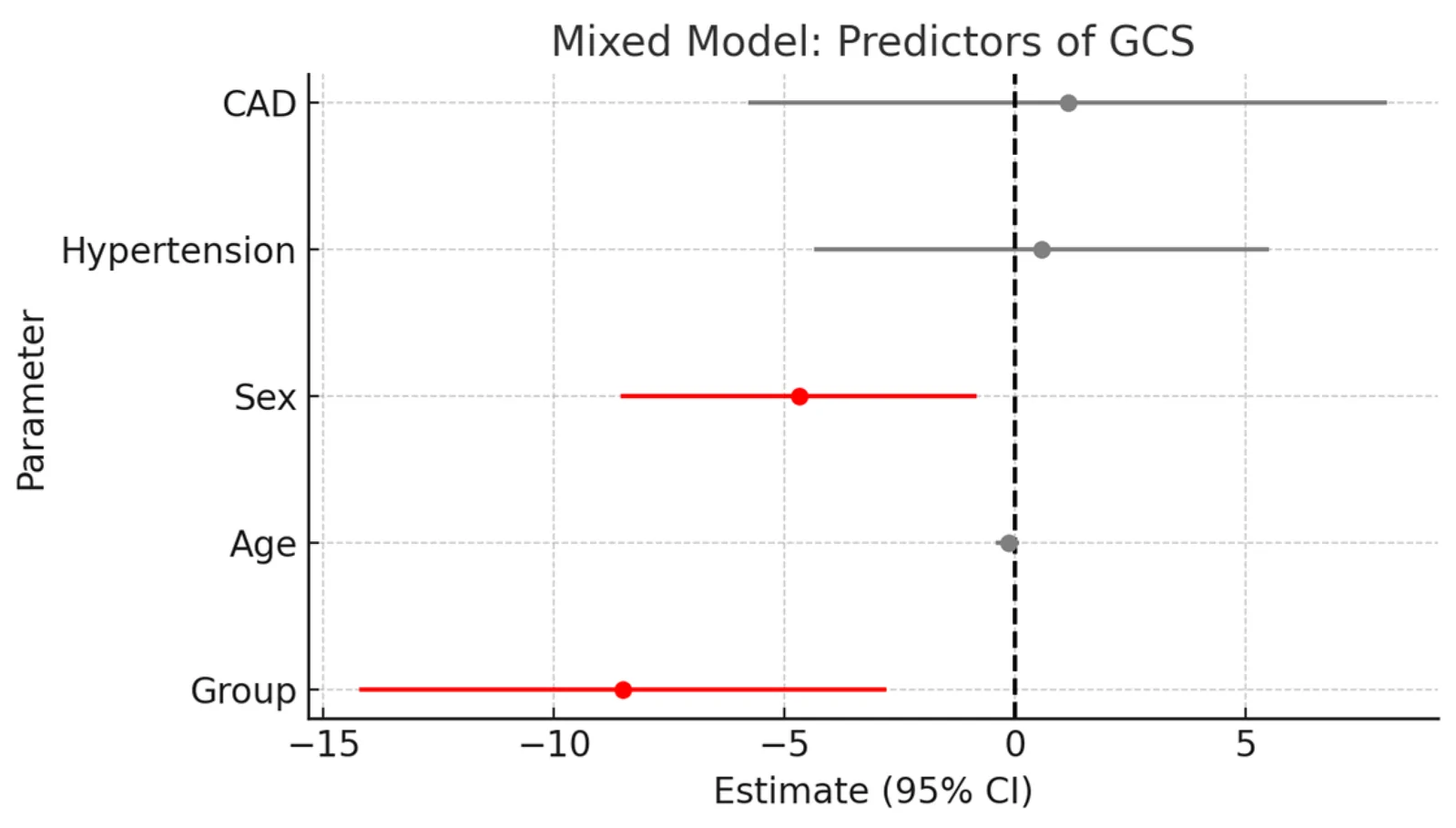
Mixed model: Dependent variable—GCS.

**Figure 3:**
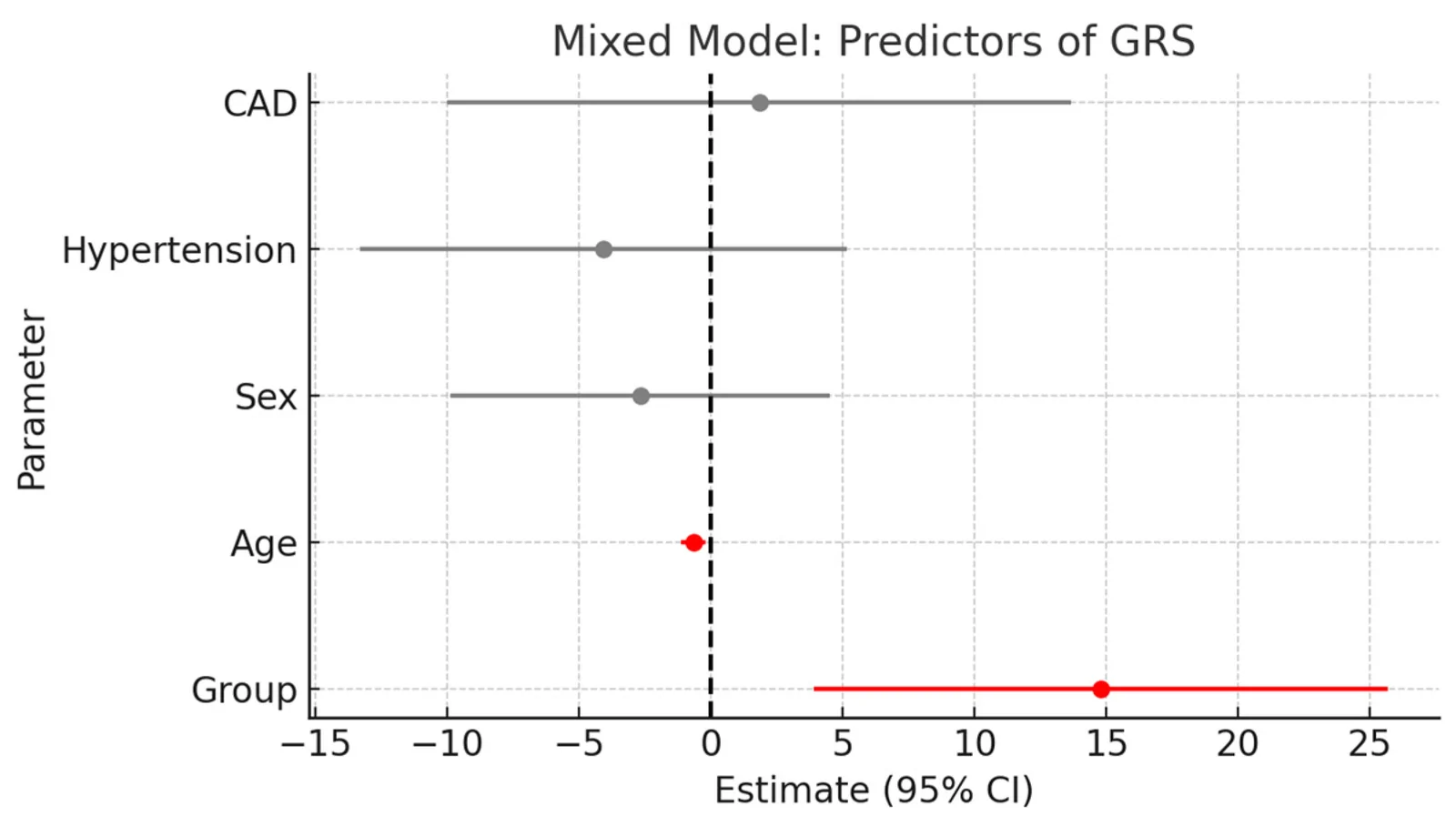
Mixed model: Dependent variable—GRS.

**Table 1:** Demographic and Clinical Data of Study Population.

Variable	SPKT (*n* = 16)	ESRD (*n* = 20)	Controls (*n* = 48)
Age (years)	39 ± 9	40 ± 10	38 ± 9
Male sex (%)	8 (50.0%)	13 (65.0%)	24 (50.0%)
BMI (kg/m^2^)	22.8 ± 2.6	24.0 ± 5.0	23.9 ± 4.0
T1DM (%)	16 (100%)	0 (0.0%)	0 (0.0%)
Peritoneal dialysis (%)	3 (18.8%)	4 (20.0%)	0 (0.0%)
Hemodialysis (%)	7 (43.8%)	15 (75.0%)	0 (0.0%)
Time on dialysis (months)	29.1 ± 54.6	49.5 ± 46.3	0.0 ± 0.0
Duration of T1DM (years)	26.1 ± 9.6	0.0 ± 0.0	0.0 ± 0.0
Hypertension (%)	15 (93.8%)	18 (90.0%)	8 (16.7%)
Coronary artery disease (%)	5 (31.3%)	4 (20.0%)	1 (2.1%)
Myocardial infarction (%)	0 (0.0%)	1 (5.0%)	0 (0.0%)
Ischemic stroke (%)	1 (6.3%)	0 (0.0%)	1 (5.0%)
Creatinine (mg/dL)	6.4 ± 2.7	8.8 ± 3.4	0.8 ± 0.2
eGFR (mL/min/1.73 m^2^)	14.9 ± 4.2	16.9 ± 8.0	87.8 ± 7.2
HbA1c (%)	7.59 ± 1.57	5.1 ± 0.44	5.24 ± 0.31

BMI = body mass index; T1DM = type 1 diabetes mellitus; eGFR = estimated glomerular filtration rate; HbA1c = hemoglobin A1c.

**Table 2:** Echocardiographic parameters of the left ventricle.

Parameter	SPKT	ESRD	Controls	*p* Value	*p* Value	*p* Value	*p* Value
					SPKT vs. Control	SPKT vs. ESRD	ESRD vs. Control
Aortic sinus (mm)	2.91 ± 0.29	2.82 ± 0.43	2.71 ± 0.38	0.113	-	-	-
LVEDD (mm)	4.41 ± 0.60	5.19 ± 0.83	4.60 ± 0.47	0.005	0.518	0.005	0.042
LVESD (mm)	2.47 ± 0.73	3.29 ± 0.76	2.85 ± 0.58	0.003	0.056	0.002	0.216
IVS (mm)	1.20 ± 0.23	1.24 ± 0.25	0.92 ± 0.17	<0.001	<0.001	1.000	<0.001
PW (mm)	1.20 ± 0.26	1.11 ± 0.26	0.92 ± 0.16	<0.001	<0.001	0.957	0.005
RWT	0.56 ± 0.17	0.44 ± 0.13	0.40 ± 0.07	0.001	<0.001	0.063	0.623
LVM (g)	195.87 ± 58.82	248.31 ± 90.16	146.87 ± 45.82	<0.001	0.023	0.399	<0.001
LVMI (g/m^2^)	110.23 ± 30.94	130.43 ± 45.71	78.04 ± 19.00	<0.001	0.001	0.919	<0.001
E (m/s)	0.94 ± 0.16	0.87 ± 0.31	0.78 ± 0.18	0.024	0.020	0.390	0.867
A (m/s)	0.80 ± 0.21	0.74 ± 0.34	0.52 ± 0.15	<0.001	<0.001	0.791	0.006
E/A ratio	1.25 ± 0.41	1.38 ± 1.01	1.63 ± 0.67	0.012	0.124	1.000	0.023
DT (ms)	155.83 ± 45.27	193.76 ± 55.56	192.61 ± 36.24	0.038	0.043	0.102	1.000
e′ sept (m/s)	0.07 ± 0.02	0.08 ± 0.02	0.11 ± 0.03	<0.001	<0.001	1.000	0.001
e′ lat (m/s)	0.10 ± 0.01	0.09 ± 0.02	0.15 ± 0.05	<0.001	0.006	1.000	<0.001
e′ mean (m/s)	0.09 ± 0.02	0.085 ± 0.02	0.13 ± 0.03	<0.001	0.002	1.000	<0.001
E/e′	11.12 ± 2.23	11.79 ± 6.70	6.25 ± 1.98	<0.001	<0.001	1.000	0.001
LVEF (%)	53.69 ± 12.52	55.60 ± 12.26	61.32 ± 4.79	0.037	0.061	1.000	0.269

Aortic sinus = sinus of Valsalva diameter; LVEDD = left ventricular end-diastolic diameter; LVESD = left ventricular end-systolic diameter; IVS = interventricular septal thickness; PW = posterior wall thickness; RWT = relative wall thickness; LVM = left ventricular mass; LVMI = left ventricular mass index; E = peak early transmitral flow velocity; A = late transmitral flow velocity; DT = deceleration time; e′ sept/lat = early diastolic mitral annular velocity (septal/lateral); LVEF = left ventricular ejection fraction.

**Table 3:** Echocardiographic parameters of the left atrium.

Parameter	SPKT	ESRD	Controls	*p* Value	*p* Value	*p* Value	*p* Value
					SPKT vs. Control	SPKT vs. ESRD	ESRD vs. Control
LA length max (cm)	5.03 ± 0.70	5.41 ± 1.22	4.57 ± 0.72	0.001	0.096	0.882	0.001
LA length pre-a (cm)	4.65 ± 0.86	4.86 ± 1.25	3.83 ± 0.69	<0.001	0.005	1.000	<0.001
LA length min (cm)	4.06 ± 0.79	4.62 ± 1.27	3.42 ± 0.72	<0.001	0.025	1.000	<0.001
LAVol max (mL)	57.06 ± 14.29	70.88 ± 35.61	36.50 ± 9.69	<0.001	<0.001	1.000	<0.001
LAVol pre-a (mL)	44.11 ± 13.85	49.43 ± 31.89	22.67 ± 7.77	<0.001	<0.001	1.000	0.001
LAVol min (mL)	23.98 ± 8.06	36.48 ± 29.85	13.92 ± 4.89	<0.001	0.001	1.000	<0.001
LAVI max (mL/m^2^)	32.02 ± 7.46	35.51 ± 14.31	19.75 ± 4.44	<0.001	<0.001	1.000	<0.001
LAVI pre-a (mL/m^2^)	24.69 ± 7.41	23.78 ± 12.89	12.32 ± 3.67	<0.001	<0.001	1.000	0.001
LAVI min (mL/m^2^)	13.45 ± 4.38	16.76 ± 10.11	7.47 ± 2.35	<0.001	<0.001	1.000	<0.001

LA = left atrium; pre-a = before atrial contraction; LAVol = left atrial volume; LAVI = left atrial volume index.

**Table 4:** Echocardiographic parameters of the right ventricle.

Parameter	SPKT	ESRD	Controls	*p* Value	*p* Value	*p* Value	*p* Value
					SPKT vs. Control	SPKT vs. ESRD	ESRD vs. Control
RVEDD basal (cm)	3.41 ± 0.51	3.59 ± 0.78	3.02 ± 0.51	0.002	0.030	1.000	0.006
RVEDD mid (cm)	3.23 ± 0.83	3.01 ± 0.59	2.70 ± 0.51	0.030	0.068	1.000	0.171
RVT (cm)	0.37 ± 0.06	0.46 ± 0.06	0.35 ± 0.07	<0.001	0.421	0.023	0.001
RVEDA (cm^2^)	18.71 ± 3.50	17.01 ± 5.26	15.28 ± 4.41	0.016	0.014	0.399	0.709
RVESA (cm^2^)	12.34 ± 3.07	10.64 ± 4.37	9.33 ± 3.21	0.006	0.004	0.170	0.854
FAC (%)	37.56 ± 21.06	38.38 ± 9.61	38.59 ± 12.08	0.345	-	-	-
TAPSE (cm)	2.54 ± 0.32	2.15 ± 0.66	2.28 ± 0.47	0.044	0.309	0.038	0.564
s’ (cm/s)	0.13 ± 0.02	0.11 ± 0.03	0.14 ± 0.02	0.001	0.535	0.193	0.001
TR (m/s)	2.15 ± 0.43	2.26 ± 0.43	1.99 ± 0.35	0.092	-	-	-
sPAP (mmHg)	18.57 ± 7.05	21.19 ± 7.69	16.60 ± 5.58	0.113	-	-	-
VCI max (cm)	1.59 ± 0.37	2.04 ± 0.52	1.73 ± 0.33	0.102	-	-	-
VCI min (cm)	0.99 ± 0.43	1.22 ± 0.55	0.93 ± 0.29	0.276	-	-	-

RVEDD = right ventricular end-diastolic diameter; RVT = right ventricular wall thickness; RVEDA = right ventricular end-diastolic area; RVESA = right ventricular end-systolic area; FAC = fractional area change; TAPSE = tricuspid annular plane systolic excursion; s’ = tissue Doppler velocity at the tricuspid annulus; TR = tricuspid regurgitation velocity; sPAP = systolic pulmonary arterial pressure; VCI = inferior vena cava.

**Table 5:** Echocardiographic parameters of the right atrium.

Parameter	SPKT	ESRD	Controls	*p* Value	*p* Value	*p* Value	*p* Value
					SPKT vs. Control	SPKT vs. ESRD	ESRD vs. Control
RAA (cm^2^)	15.64 ± 3.02	16.78 ± 6.40	13.19 ± 2.36	0.004	0.024	1.000	0.023
RAVol (mL)	44.37 ± 15.55	50.37 ± 33.06	33.13 ± 9.99	0.005	0.026	1.000	0.028
RAVI (mL/m^2^)	24.76 ± 6.99	23.67 ± 8.77	18.04 ± 4.39	0.001	0.003	1.000	0.030

RAA = right atrial area; RAVol = right atrial volume; RAVI = right atrial volume index.

**Table 6:** Left Ventricular Strain Analysis.

Parameter	SPKT	ESRD	Controls	*p* Value	*p* Value	*p* Value	*p* Value
					SPKT vs. Control	SPKT vs. ESRD	ESRD vs. Control
GLS basal (%)	13.07 ± 2.69	14.68 ± 4.87	19.78 ± 1.89	<0.001	<0.001	0.377	<0.001
GLS mid (%)	15.97 ± 3.09	17.18 ± 5.56	22.85 ± 2.24	<0.001	<0.001	0.480	<0.001
GLS apical (%)	13.07 ± 2.69	14.65 ± 4.87	19.79 ± 1.88	<0.001	<0.001	0.431	<0.001
GLS average (%)	10.69 ± 2.42	12.72 ± 4.37	17.19 ± 1.68	<0.001	<0.001	0.316	<0.001
GCS (%)	10.73 ± 3.67	19.27 ± 8.17	20.09 ± 7.18	<0.001	<0.001	0.002	1.000
GRS (%)	13.37 ± 13.30	25.19 ± 12.75	30.67 ± 12.96	<0.001	<0.001	0.047	0.524

GLS = global longitudinal strain; GCS = global circumferential strain; GRS = global radial strain.

**Table 7:** Left atrial strain and strain rates.

Parameter	SPKT	ESRD	Controls	*p* Value	*p* Value	*p* Value	*p* Value
					SPKT vs. Control	SPKT vs. ESRD	ESRD vs. Control
LA PACS	18.54 ± 7.67	11.57 ± 4.22	16.91 ± 5.67	0.002	1.000	0.009	0.003
LA PALS	26.69 ± 8.69	25.98 ± 11.04	39.29 ± 11.56	<0.001	0.001	1.000	0.001
LA SRs	1.25 ± 0.36	1.17 ± 0.42	1.72 ± 0.49	<0.001	0.003	1.000	<0.001
LA SRe	1.02 ± 0.64	1.22 ± 0.67	2.17 ± 0.91	<0.001	<0.001	1.000	<0.001
LA SRa	1.65 ± 0.56	1.61 ± 0.57	2.13 ± 0.71	0.013	0.068	1.000	0.048

LA PACS = left atrial pre-atrial contraction strain; LA PALS = left atrial peak atrial longitudinal strain; SRs = strain rate during systole; SRe = strain rate during early diastole; SRa = strain rate during atrial contraction.

**Table 8:** Right ventricular strain analysis.

Parameter	SPKT	ESRD	Controls	*p* Value	*p* Value	*p* Value	*p* Value
					SPKT vs. Control	SPKT vs. ESRD	ESRD vs. Control
RV GLS (%)	18.33 ± 7.41	16.48 ± 6.41	19.28 ± 4.75	0.315	-	-	-
RV GLS free wall (%)	23.98 ± 9.25	20.07 ± 8.93	22.77 ± 6.86	0.281	-	-	-
RV GLS endocardial (%)	21.14 ± 8.29	19.33 ± 6.69	22.12 ± 5.43	0.318	-	-	-
RV GLS midmyocardial (%)	18.33 ± 7.41	16.25 ± 6.51	19.23 ± 4.79	0.280	-	-	-
RV GLS epicardial (%)	15.93 ± 7.09	14.38 ± 6.34	17.41 ± 4.40	0.307	-	-	-

RV GLS = right ventricular global longitudinal strain.

**Table 9:** Right atrial strain and strain rates.

Parameter	SPKT	ESRD	Controls	*p* Value	*p* Value	*p* Value	*p* Value
					SPKT vs. Control	SPKT vs. ESRD	ESRD vs. Control
RA PACS (%)	19.67 ± 14.64	14.69 ± 9.65	13.93 ± 5.97	0.717	-	-	-
RA PALS (%)	31.07 ± 17.75	28.94 ± 18.98	33.89 ± 12.96	0.229	-	-	-
RA SRs (1/s)	1.64 ± 0.67	1.43 ± 0.74	1.59 ± 0.50	0.396	-	-	-
RA SRe (1/s)	1.49 ± 0.88	1.91 ± 0.96	1.66 ± 0.67	0.431	-	-	-
RA SRa (1/s)	0.89 ± 0.83	0.98 ± 0.44	1.35 ± 0.58	0.006	0.016	1.000	0.071

RA = right atrium; PACS = peak atrial contraction strain; PALS = peak atrial longitudinal strain; SRs = systolic strain rate; SRe = early diastolic strain rate; SRa = late diastolic strain rate.

## Data Availability

The data presented in this study are available on request.
